# Meta-Analysis of the Effect of Overexpression of Dehydration-Responsive Element Binding Family Genes on Temperature Stress Tolerance and Related Responses

**DOI:** 10.3389/fpls.2018.00713

**Published:** 2018-05-29

**Authors:** Chao Dong, Yuanchun Ma, Dan Zheng, Michael Wisniewski, Zong-Ming Cheng

**Affiliations:** ^1^College of Horticulture, Nanjing Agricultural University, Nanjing, China; ^2^United States Department of Agriculture – Agricultural Research Service (USDA-ARS), Kearneysville, WV, United States; ^3^Department of Plant Sciences, The University of Tennessee, Knoxville, Knoxville, TN, United States

**Keywords:** *DREB*, meta-analysis, temperature stress, overexpression, stress-related parameters

## Abstract

Dehydration-responsive element binding proteins are transcription factors that play a critical role in plant response to temperature stress. Over-expression of *DREB* genes has been demonstrated to enhance temperature stress tolerance. A series of physiological and biochemical modifications occur in a complex and integrated way when plants respond to temperature stress, which makes it difficult to assess the mechanism underlying the *DREB* enhancement of stress tolerance. A meta-analysis was conducted of the effect of *DREB* overexpression on temperature stress tolerance and the various parameters modulated by overexpression that were statistically quantified in 75 published articles. The meta-analysis was conducted to identify the overall influence of *DREB* on stress-related parameters in transgenic plants, and to determine how different experimental variables affect the impact of *DREB* overexpression. Viewed across all the examined studies, 7 of the 8 measured plant parameters were significantly (*p* ≤ 0.05) modulated in *DREB*-transgenic plants when they were subjected to temperature stress, while 2 of the 8 parameters were significantly affected in non-stressed control plants. The measured parameters were modulated by 32% or more by various experimental variables. The modulating variables included, acclimated or non-acclimated, type of promoter, stress time and severity, source of the donor gene, and whether the donor and recipient were the same genus. These variables all had a significant effect on the observed impact of *DREB* overexpression. Further studies should be conducted under field conditions to better understand the role of *DREB* transcription factors in enhancing plant tolerance to temperature stress.

## Introduction

Concern over temperature stress has become exacerbated due to climate change and is a major research topic of plant scientists worldwide. Temperature stress limits plant growth and reduces yield, based on the optimum temperature limits of each species (Kourosh and Charles, 2013). Temperature stress includes both high temperature (HT) stress and low temperature (LT) stress. LT stress comprises chilling stress (<20°C) and freezing stress (<0°C) that result in poor plant growth and even death (Zhuang et al., 2015). A variety of genes has been shown to be induced by LT in many economically important crops, and notably among the induced genes are CBF (C-repeat binding factor) and DREB (dehydration responsive element binding) transcription factors.

*CBF/DREB*1, one of the first discovered members of the *DREB* gene family, induce the expression of cold-regulated (*COR*) genes in *Arabidopsis thaliana* and its overexpression has been shown to enhance freezing and drought tolerance in transgenic plants (Jaglo-Ottosen et al., 1998; Liu et al., 1998). Studies of the CBF/DREB pathway have revealed many additional cold-induced genes, including other transcription factors whose function has also been characterized (Lata and Prasad, 2011). Several different DREB transcription factors have been over-expressed in agriculturally important crops, such as rice and wheat, and in model plants, such as *Arabidopsis* and *Nicotiana* (Kasuga et al., 2004; Oh et al., 2005; Ito et al., 2006; Morran et al., 2011). Collectively, these studies indicate that different DREB transcription factors are specifically responsive to drought stress, salt stress, heat stress, low-temperature stress, and exogenous ABA, and have the potential to be used to improve plant stress tolerance due their ability to regulate the expression of downstream genes associated with stress tolerance (Wang et al., 2008; Kidokoro et al., 2015).

The effect of temperature stress on the growth of plants is complex, and many physiological and biochemical processes are affected, through the up and or down regulation of a variety of different genes, multiple signaling pathways, and multiple gene products. Many DRE-binding transcription factors respond to temperature stress and DREBs have been shown to induce CRT/DRE-regulated downstream target COR genes (Akhtar et al., 2012). The complete signaling pathways and regulatory mechanisms responsible for plant responses to temperature stress are still incomplete and require additional study. A large body of research has demonstrated that plant response to temperature stress is a quantitative and complex trait controlled by many genes (Xu, 2012). This makes it difficult to discern the molecular mechanisms underlying temperature stress responses and determine which plant phenotypic traits are due solely to the overexpression of *DREBs*.

When a plant is subjected to temperature stress, physiological and biochemical reactions occurs within cells, which result in distinct phenotypic changes in a plant. Therefore, in studies on the impact of the over expression of *DREB* on stress tolerance in plants, a variety of variables are affected and used as indicators. Gene recipients and donors of *DREB* transcription factor genes have been both dicots and monocots. Stress duration and stress severity have also varied greatly in the reported studies. Different promoters have been used to drive the expression of *DREB* genes. Exposure to cold acclimating conditions also can improve the freezing tolerance of transgenic plants relative to wild-type plants. Collectively, the studies have utilized a wide variety of variables to induce, evaluate, and measure increases in the freezing tolerance of transgenic plants, making the interpretation of the reported results difficult and at times problematic.

A meta-analysis was performed on the impact of cation/proton antiporter 1 (*CPA*1) genes which included numerous moderator variables in the analyses (Ma et al., 2017). Meta analysis utilizes statistical methods to analyze a collection of research data obtained from many independent experiments and studies in order to determine the impact of the various variables on a variety of phenotypic responses. A benefit of a meta-analysis is that it increases the reliability of specific results by increasing the sample size and by considering the variability inherent in the reported results (Borenstein et al., 2009). In the present study, a meta-analysis was conducted on the over-expression of *DREB* to determine the impact of various variables, such as donor and recipient species, on specific phenotypic traits, such as photosynthesis and plant growth. Meta-analysis allows one to determine the overall direction and magnitude of plant responses to temperature stress. Eight different moderator variables were examined that can potentially affect the magnitude of the effect of *DREB* overexpression on plant tolerance to LT stress. An attempt was made to address the following questions:

(1) What is the overall impact of *DREB* overexpression on plant response to LTs in transgenic plants, across all studies?
(1) Is the impact of *DREB* overexpression more significant in temperature stressed plants than in non-stressed plants?
(1) How have experimental variables affected the magnitude of the impact of plant response to *DREB* overexpression?

The results of the conducted meta-analysis can help to determine which plant characteristics have been most affected by *DREB* overexpression and help to identify research areas that will enable researchers to better understand the role of *DREB* transcription factors in enhancing plant tolerance to LT stress.

## Materials and Methods

### Data Collection

The research studies included in the meta-analysis were identified through a systematic search of 12 electronic databases, incorporating a full range of measured indicators, plant species and experimental designs, identified and selected using ISI Web of Science and Endnote search tools (additional search details provided in **Supporting Information 1**). The literature search was performed in April – September 2016 using a combination of keywords, including ‘CBF transcription factors,’ DREB transcription factors, ‘CBF,’ ‘DREB,’ cold^∗^, stress^∗^, transgenic, and overexpression (see **Supporting Information 1**). A total of 455 unique studies were obtained, of which 70 were suitable for inclusion in the meta-analysis. The literature cited sections of these articles were also examined to identify further potentially relevant articles. This resulted in the inclusion of five additional research articles. In total, 75 articles, written in English and Chinese spanning 18 years, on the overexpression of *DREB* in transgenic plants were included in the meta-analysis. Citations and details of the included studies are provided in **Supporting Information 1**.

Multiple treatments within a single article were considered as independent observations and represent individual studies in a meta-analysis (Rosenthal and Wolf, 1986; Marczak et al., 2007). Means and sample sizes were gathered for both the treatment and controls from each of the individual studies. When sample size was given as a range, the minimum value was used in the meta-analysis. Measurements were taken at every time point or stress levels were collected from studies that included data covering several treatment levels or time courses. Software (GetData Graph Digitizer) was used to digitize data when data were only available in figures^1^.

### Effect Size and Moderator

**FIGURE 1:**
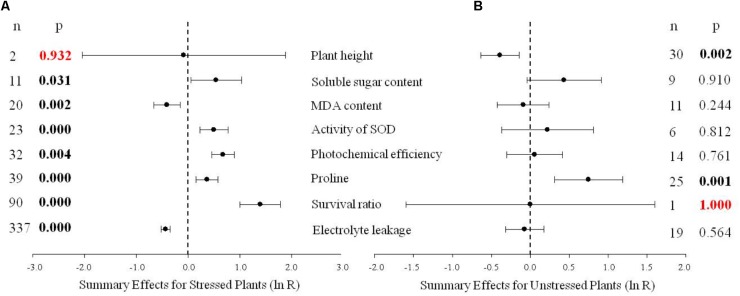
Weighted summary effect sizes (natural log of the transformed/control plant response ratios, ln *R*). Horizontal bars extending from the closed circle represent 95% confidence intervals (CIs). The dashed vertical line indicates effect size = 0. Summary effects were analyzed in plants exposed to temperature stress **(A)** and non-stress conditions **(B)**. *n*, the number of studies in each summary effect. *P* ≤ 0.05 indicates a summary effect that is significantly different from zero (same for **Figures 2**–**10**).

A meta-analysis was conducted on the response of several variables in control and *DREB* transgenic plants that were subjected to a low-temperature stress (**Figure 1**). Meta analysis was used to compare the effect size in each of the studies, using the natural log of the response ratio (ln *R*) of the means in transgenic plants (TC) to the means in non-transgenic plants (NC) (Hedges et al., 1999);

$$InR=In\,\;Y_{TC}/Y_{NC}$$

An ln *R*-value below 0 indicates a TC-induced (transgenic plants-induced) decrease in the response of the measured parameter, while a value above 0 indicates a TC-induced increase in the response of the measured parameter. A value of 0 indicates that *DREB* over-expression had a neutral effect on the measured parameter. The ln *R*-value is widely used in meta-analyses to statistically quantify the effects of variables and treatments in biological science and indicate direct biological significance (Lehmann et al., 2012; Augè et al., 2014; Ma et al., 2017). Treatment and control comparison must be made when individual specimens are in same growth period and the same growth conditions, to avoid potential confounding effects and be considered valid (Slattery et al., 2013).

Moderator variables were used to test for heterogeneity within the effect sizes. These consisted of stress severity and stress duration, and several plant attributes, including the type of promoter used, taxonomic class (monocot or dicot, or other) and genus of the gene donor and recipient, whether the donor and recipient were the same genus, and growth condition: (non-acclimated or cold-acclimated). To reduce bias, each moderator variable needed to include at least two levels (categories), and the data within each level was required to be sourced from at least three studies, within ≥2 articles. A categorical level that did not meet the assumptions required for meta-analysis, it was grouped into a level analyzed as “other.” The “other,” however, level was also required, to contain data obtained from at least three studies within ≥2 articles.

### Meta-Analysis

A random effects model analysis, which allows true effect sizes to vary, was used to conduct the meta-analysis. All analyses were conducted using Comprehensive Meta-Analysis (CMA) software (Version 3.0, Biostat, Englewood, NJ, United States; 2016), which creates forest and funnel plots, and computes the rank correlation. Individual studies were weighted using a non-parametric variance equation:

$$VInR=(n_{TC}+n_{NC})/\left(n_{TC}*n_{NC}\right)$$

where *n*_TC_ and *n*_NC_ are the sample sizes of the TC (transgenic plants) and NC (non-transgenic plants) treatments and *V*ln *R* is the variance of the natural log of the response ratio (Borenstein et al., 2009). The summary effect was considered significant at *P* < 0.05 when its confidence intervals (CI) did not bracket zero. The effect of a categorical variable was considered significant when the 95% CIs did not include zero.

The *Q* statistic (a measure of weighted squared deviations) was used to evaluate whether or not data exhibit heterogeneity and *I*^2^ (a descriptive index that estimates the ratio of true heterogeneity) was used to quantify heterogeneity across the observed effect sizes (Higgins and Thompson, 2002; Borenstein et al., 2009). A *p*-value for the *Q*-test below 0.1 indicates significant heterogeneity in the summary effect sizes (Iacovelli et al., 2014). A *I*^2^ value of 0% indicates no true heterogeneity, while larger values reflect a larger proportion of the observed variation. A common among-study variance across moderator subgroups was assumed when the 95% CI of effect size overlapped zero and its *p*-value was less than 0.05.

Although a search strategy was used to identify and access literature from several sources to avoid publication bias, whether this bias exists is not known. Therefore, funnel plots were used to visually identify the presence of publication bias. Egger’s linear regression and Begg and Mazumdar rank (Kendall) correlation (Begg and Mazumdar, 1994; Sterne and Egger, 2006) were also used to assess potential publication bias. Additional details and information are provided in our previous paper (Dong et al., 2017).

## Results

### Effect of *DREB* Overexpression in Transgenic Plants

**Figure 1** indicates eight summary effect sizes of TC/NC response ratios in plants subjected to a cold stress and in non-stressed plants. Results were based on 31 species within the 570 studies contained in the 75 publications used in the meta-analysis (**Supporting Information 1**-Studies). *DREB* genes obtained from *Arabidopsis* were the most highly studied (304 studies). Among gene donor plant types, the greatest amount of data was obtained from dicotyledonous plants (509 studies), and dicotyledonous plants also represented the greatest number (508 studies) in the gene recipient category. Fifteen species of recipient plants were studied and *A. thaliana* was the most represented (238 studies). The data set included eight promoters, with CaMV35S being the most utilized promoter (183 studies).

**FIGURE 2:**
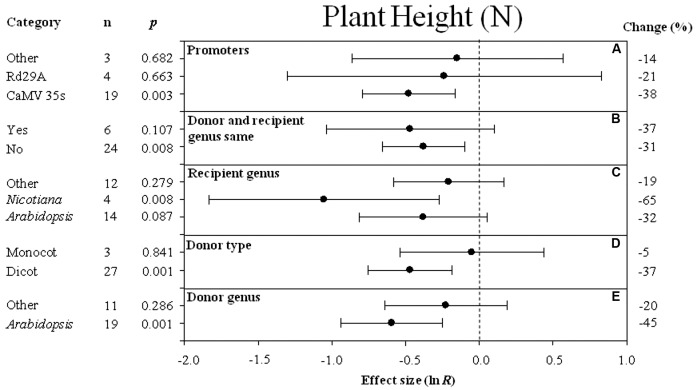
Effect sizes (as natural logs, ln *R*) of overexpression of *DREB* with 95% CI on plant height. Effect sizes were analyzed in non-stressed plants and are represented as the effect size (closed circle) with 95% CI (horizontal bar). The dashed vertical line indicates an effect size = 0. The effect of five moderator variables **(A–E)** on the magnitude of the treatment effect resulting from overexpression of *DREB* is presented. Category indicates the components comprising each moderator. Percent change is listed on the right and indicates raw percentage change increase in plant height induced by *DREB* overexpression.

**FIGURE 3:**
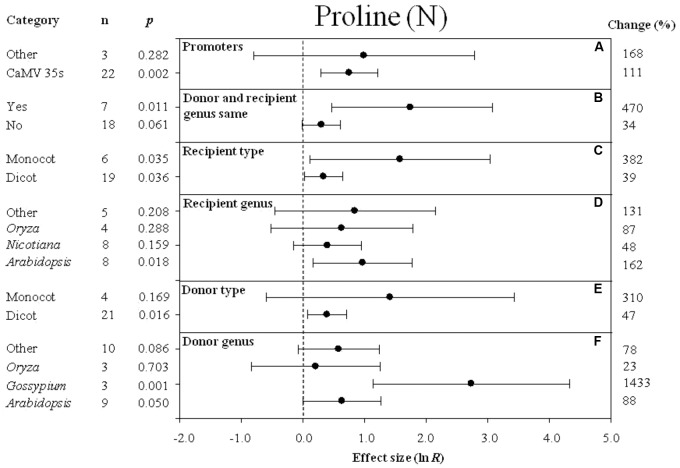
Effect sizes (as natural logs, ln *R*) of overexpression of *DREB* with 95% CIs on proline content. Effect sizes were analyzed in non-stressed plants. The dashed vertical line indicates an effect size = 0. The effect of six moderator variables **(A–F)** on the magnitude of the treatment effect is presented. Category indicates the components comprising each moderator. Percent change is listed on the right and indicates raw percentage change increase in plant height induced by *DREB* overexpression.

**FIGURE 4:**
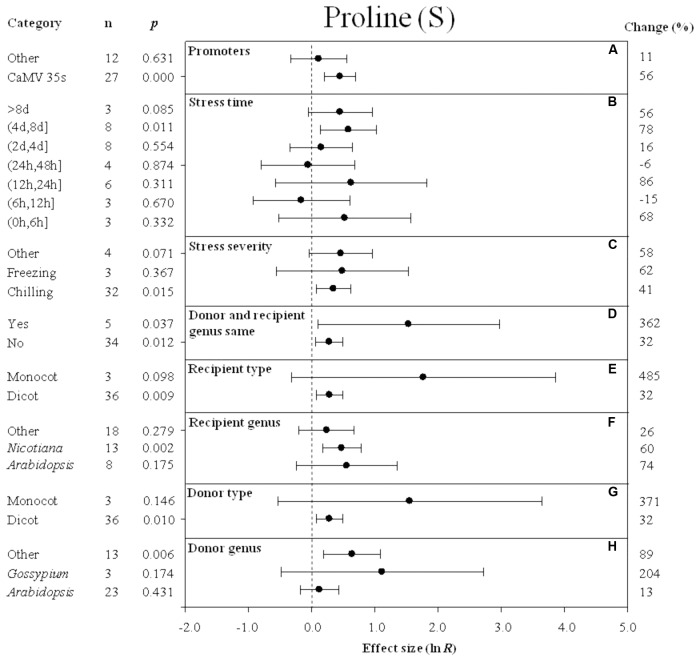
Effect sizes (as natural logs, ln *R*) of overexpression of *DREB* with 95% CIs on proline content. Effect sizes were analyzed in plants exposed to temperature stress. The dashed vertical line indicates an effect size = 0. The effect of eight moderator variables **(A–H)** on the magnitude of the treatment effect is presented. Category indicates the components comprising each moderator. Percent change is listed on the right and indicates raw percentage change in proline content induced by *DREB* overexpression.

**FIGURE 5:**
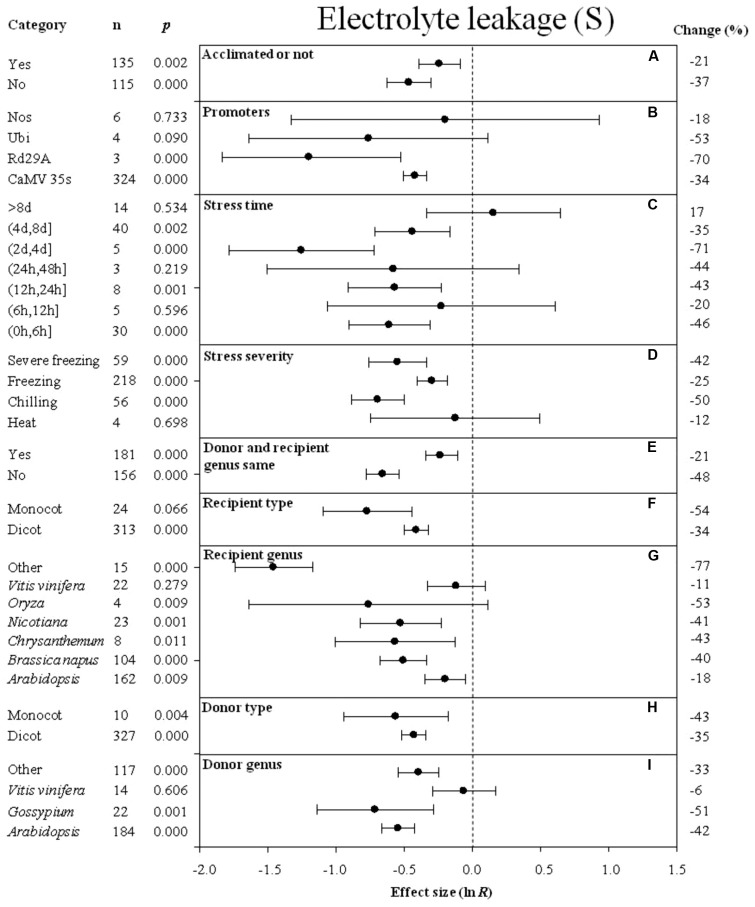
Effect sizes (as natural logs, ln *R*) of overexpression of *DREB* with 95% CIs on electrolyte leakage. Effect sizes were analyzed in plants subjected to temperature stress. The dashed vertical line indicates effect size = 0. The effect of nine moderator variables **(A–I)** on the magnitude of the treatment effect is presented. Category indicates the components comprising each moderator. Percent change is listed on the right and indicates raw percentage change in electrolyte leakage induced by *DREB* overexpression.

**FIGURE 6:**
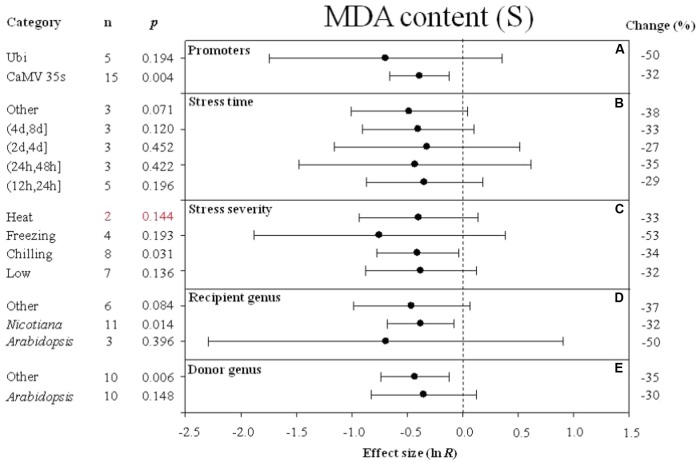
Effect sizes (as natural logs, ln *R*) of overexpression of *DREB* with 95% CIs on MDA content. Effect sizes were analyzed in plants subjected to temperature stress. The dashed vertical line indicates effect size = 0. The effect of five moderator variables **(A–E)** on the magnitude of the treatment effect is presented. Category indicates the components comprising each moderator. Percent change is listed on the right and indicates raw percentage change in MDA content induced by *DREB* overexpression.

**FIGURE 7:**
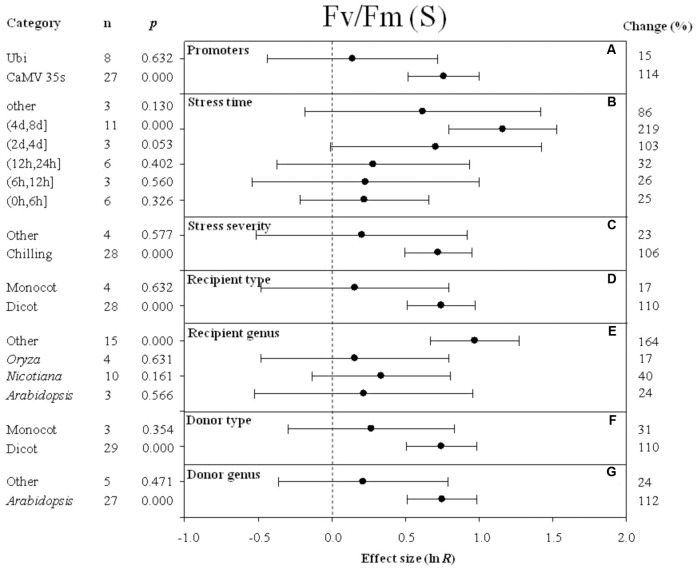
Effect sizes (as natural logs, ln *R*) of overexpression of *DREB* with 95% CIs on *F*_v_/*F*_m_. Effect sizes were analyzed in plants subjected to temperature stress. The dashed vertical line indicates effect size = 0. The effect of eight moderator variables **(A–G)** on the magnitude of the treatment effect is presented. Category indicates the components comprising each moderator. Percent change is listed on the right and indicates raw percentage change in *F*_v_/*F*_m_ induced by *DREB* overexpression.

**FIGURE 8:**
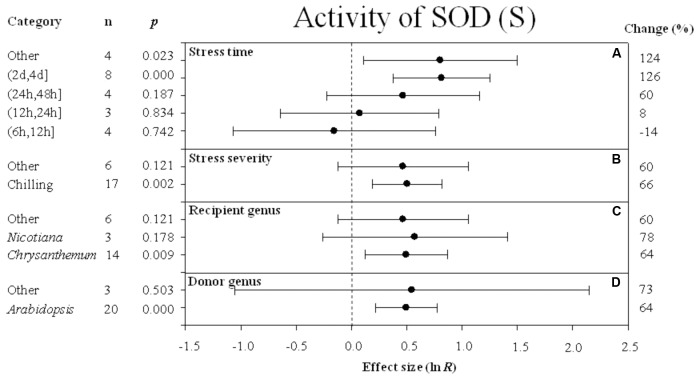
Effect sizes (as natural logs, ln *R*) of overexpression of *DREB* with 95% CIs on activity of SOD. Effect sizes were analyzed in plants subjected to temperature stress. The dashed vertical line indicates effect size = 0. The effect of four moderator variables **(A–D)** on the magnitude of the treatment effect is presented. Category indicates the components comprising each moderator. Percent change is listed on the right and indicates raw percentage change in RWC induced by *DREB* overexpression.

**FIGURE 9:**
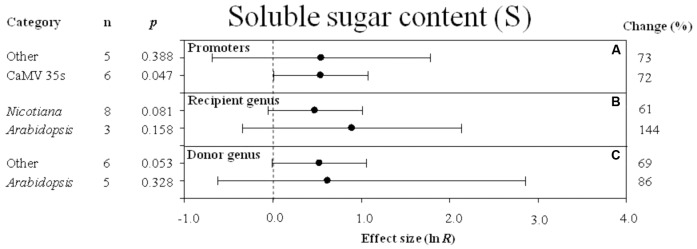
Effect sizes (as natural logs, ln *R*) of overexpression of *DREB* with 95% CIs on soluble sugar content. Effect sizes were analyzed in plants subjected to temperature stress. The dashed vertical line indicates effect size = 0. The effect of three moderator variables **(A–C)** on the magnitude of the treatment effect is presented. Category indicates the components comprising each moderator. Percent change is listed on the right and indicates raw percentage change in soluble sugar content induced by *DREB* overexpression.

**FIGURE 10:**
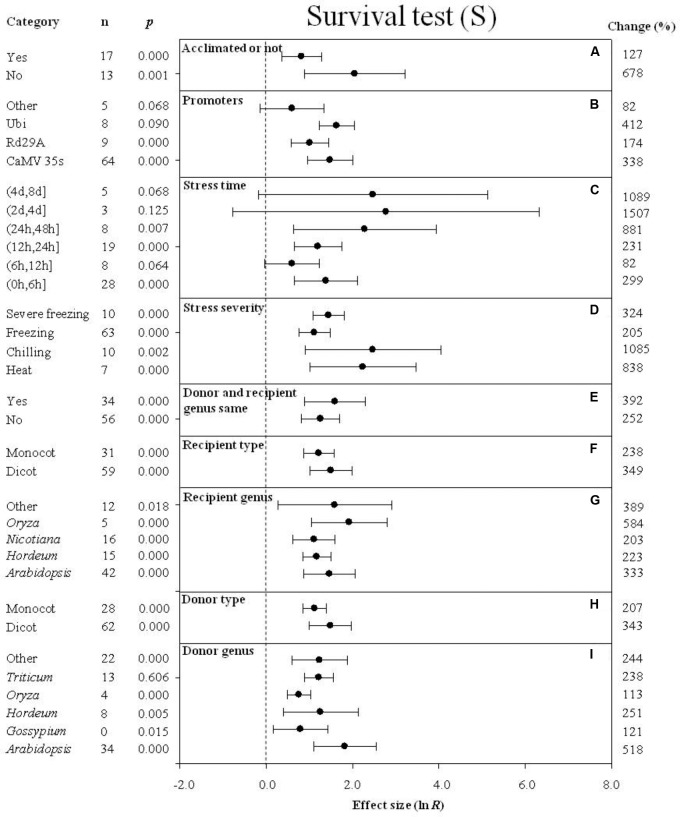
Effect sizes (as natural logs, ln *R*) of overexpression of *DREB* with 95% CIs on survival. Effect sizes were analyzed in plants subjected to temperature stress. The dashed vertical line indicates effect size = 0. The effect of nine moderator variables **(A–I)** on the magnitude of the treatment effect is presented. Category indicates the components comprising each moderator. Percent change is listed on the right and indicates raw percentage change in the survival induced by *DREB* overexpression.

Natural logs of summary effect sizes were graphically illustrated in forest plots (**Figures 1**–**10**), and the rate of change of the summary effect on physiological and growth indexes in non-stressed and cold-stressed plants resulting from the overexpression of *DREB* in transgenic plants are summarized in **Table 1**. A total of 7 out of the 8 measured plant parameters were significantly affected when plants were subjected to cold stress (**Figure 1A**: *p* ≤ 0.05, 95% CIs do not cross zero), increasing or decreasing the measured values by 34% or more (**Table 1**). The most affected parameter was survival, being 303% more greatly affected in stressed than in non-stressed plants. Photochemical efficiency and soluble sugar content in TC plants increased by 96 and 72%, respectively, relative to NC plants. Proline and SOD (superoxide dismutase) activity also increased significantly in transgenic plants. Overexpression of a *DREB* gene in transgenic plants had a significant negative effect on electrolyte leakage and MDA (malondialdehyde), being 35 and 34%, respectively, relative to non-transgenic plants. In contrast, only 2 of the 8 plant parameters were significantly affected (*p* ≤ 0.05) by *DREB* overexpression in non-stressed plants (**Figure 1B**). The effect on proline levels was significantly increased by 111% in transgenic plants, relative to wild-type, non-transformed plants, while a significant negative effect on plant height was evident in transgenic plants, relative to non-transgenic, control plants.

**Table 1:** Heterogeneity statistics for the eight summary effect sizes under stress and non-stress conditions.

Summary effect size	Qt	*p*_hetero_	*I*^2^ (%)	TC-induced change (%)
Electrolyte leakage (S)	285.636	0.978	0.0	**-35**
Survival test (S)	72.107	0.904	0.0	**303**
Proline (S)	37.945	0.472	0.0	**44**
*F*_v_/*F*_m_ (S)	28.369	0.602	0.0	**96**
Activity of SOD (S)	17.158	0.755	0.0	**65**
MDA content (S)	2.938	1.000	0.0	**-34**
Soluble sugar content (S)	0.971	1.000	0.0	**72**
Plant height (S)	0	1.000	0.0	**-**8
Electrolyte leakage (N)	1.916	1.000	0.0	**-**7
Survival test (N)	0		0.0	0
Proline (N)	23.652	0.482	0.0	**111**
*F*_v_/*F*_m_ (N)	0.371	1.000	0.0	6
Activity of SOD (N)	0.257	0.998	0.0	25
MDA content (N)	1.152	1.000	0.0	**-**9
soluble sugar content (N)	1.721	0.998	0.0	54
Plant height (N)	24.442	0.707	0.0	**-32**


**Qt, total heterogeneity; p_*hetero*_, probability that Qt was due entirely to sampling error and not to variation among true effects; I^*2*^, percentage of heterogeneity due to variation among true effects; changes to summary effects caused by foreign DREB genes, transformed from ln R to raw percentages for TC-induced changes, bold text signifies change was significant (p ≤ 0), with positive values indicating TC-induced promotion and negative values TC-induced inhibition. (S), summary effects for stressed plants; (N), summary effects for non-stressed plants.**

### Heterogeneity and Moderator Analysis

No significant heterogeneity was observed in any of the summary effects (*p*_hetero_ values > 0.10, **Table 1**). *I*^2^ = 0.0 for each of the 8 summary effects. Borenstein et al. (2009) stated that a significant *p*_hetero_ value indicates statistical heterogeneity. A *p*_hetero_ value > 0.1 is insufficient to demonstrate that true effects are consistent with summary effects. A small sample size or large differences in experimental conditions are conditions that may lead to a lack of significance. Therefore, a random effect model was selected to conduct a subgroup analysis of different variables.

Forest plots graphically illustrate the summary effect. The CIs in a forest plot are used to evaluate significant differences in moderator levels, with respect to their overlap with a zero value. A moderator analysis was conducted on nine summary effects that were significantly affected by *DREB* overexpression to understand the effect of the different categories of variables or different levels within a single variable on the summary effects. **Figures 2**–**10** illustrate the impact of moderator levels on the summary effects that exhibited the most sensitivity to an influence by the moderators.

### The Effect of Each Moderator on Transgenic Plant Height Under Non-stressed Conditions

The effect of transformation on reducing plant height in non-stressed plants was significant when *DREB* expression was driven by the CaMV35S promoter (**Figure 2A**). Transformation had a slightly negative effect whether donors and recipients were of the same species or not (-37 and -31%, respectively) (**Figure 2B**). *Nicotiana tabacum* appeared to be the species most affected by *DREB* overexpression in regard to plant height (**Figure 2C**). Transformation had a significant negative effect on plant height when the *DREB* genes used to generate the transgenic plants came from a dicot (**Figure 2D**). The effect of *Arabidopsis*, as a donor genus, on plant height was two times larger than other donor genera (**Figure 2E**).

### Effect of Subgroup Within Each Moderator on Proline Levels Measured in Transgenic Plants Subjected to Non-stressed and Cold-Stressed Conditions

The effect of transformation on the proline levels in transgenic plants overexpressing a *DREB* gene and subjected to non-stressed or cold-stressed conditions was not consistent among the different promoters (a moderator). Although *DREB* expression had a positive effect on proline levels, the effect on proline content in transgenic, non-stressed plants was lower when *DREB* gene expression was driven by the CaMV35s promoter (**Figure 3A**). In transgenic cold-stressed plants, it was higher when *DREB* gene expression was driven by the CaMV35s promoter than when driven by other promoters (**Figure 4A**). However, a significant effect was observed only in the transgenic plants where *DREB* gene expression was driven by the CaMV35s promoter (**Figures 3A**, **4A**). The effect of transformation on the proline levels in both non-stressed and cold-stressed transgenic plants was at least 14× greater when the donor and recipient genera were the same genus, compared to when the donor and recipient were of different genera (**Figures 3B**, **4B**). Monocots, as a recipient-type moderator, had a 10× greater positive effect on proline levels than dicots in both non-stressed and cold stress transgenic plants (**Figures 3C**, **4E**). This trend was also evident in the moderator, donor-type plants, where monocots had a 6× greater positive effect on proline levels than dicots in non-stressed transgenic plants, and a 16× greater effect than dicots in cold-stressed transgenic plants (**Figures 3E**, **4G**). *Arabidopsis* as a donor genus and a recipient genus, had a significantly positive effect on proline levels when transgenic plants were subjected to non-stressed conditions, however, when transgenic plants were subjected to a cold stress, it only had a slight effect on proline levels (**Figures 3D,F**, **4F,H**). *Gossypium* as a donor genus, had the greatest positive effect (1433% increase) on proline levels when transgenic plants were grown under non-stressed conditions (**Figure 3F**).

When the duration of the cold-stress was 4–8 days, the effect of *DREB* gene overexpression on the level of proline in transgenic plants was significantly increased by 78%. When transgenic plants were subjected to a cold-stress treatment for 6–12 or 24–48 h, proline levels were slightly reduced by -15 and -6% (**Figure 4B**), relative to non-transgenic plants. Stress severity (chilling or freezing stress) also affected the level of proline in transgenic plants when plants were subjected to chilling stress conditions (**Figure 4C**).

### Electrolyte Leakage and MDA Content

The degree of the impact of several moderators on the effect of *DREB* overexpression on electrolyte leakage in cold-stressed plants is presented in **Figure 5**. A greater negative effect on electrolyte leakage was observed when plants were not acclimated prior to the cold stress than when plants were acclimated prior to the cold stress (**Figure 5A**). A lower level of electrolyte leakage was obtained in transgenic plants when rd29A or Ubi was used as a promoter than when CaMV35s was used as a promoter to drive *DREB* expression (**Figure 5B**). A long-term duration of stress resulted in a slightly larger degree of electrolyte leakage in transgenic, cold-stressed plants than when a shorter durations of stress exposure was used in the experiment (**Figure 5C**). The effect of *DREB* overexpression on the electrolyte leakage was about two times lower when plants were subjected to a chilling and severe freezing stress than when plants were subjected to a moderate freezing stress (**Figure 5D**). When the *DREB* donor and recipient were from different genera, the effect of overexpression on electrolyte leakage was two times lower than when the donor and recipient were from the same genus (**Figure 5E**). The effect of *DREB* overexpression on the electrolyte leakage was more reduced when the recipient and donor plants were monocots (**Figures 5F,H**) than when they were dicots. *Vitis vinifera* as a donor genus and a recipient genus, had the least effect on electrolyte leakage, compared to other genera that significantly reduced electrolyte leakage (**Figures 5G,I**).

Similar to the effect of promoter (as a moderator) on electrolyte leakage, the effect on MDA content was significantly reduced when *DREB* expression was driven by the CaMV 35s promoter (**Figure 6A**). The effect of *DREB* overexpression on MDA content was slightly reduced, regardless of the duration of the stress (**Figure 6B**). The effect of transformation on MDA was significantly reduced when transgenic plants were subjected to a chilling stress (**Figure 6C**). The effect of transformation on MDA was reduced to a greater degree when the recipient of the *DREB* gene was *Arabidopsis* than when the recipients were a genus other than *Arabidopsis* (**Figure 6D**). In contrast, the effect of lowering MDA content was greater when the genus of the *DREB* gene donor was a genus other than *Arabidopsis* (**Figure 6E**).

### Photosynthesis

The effect of *DREB* overexpression on the increase in *F*_v_/*F*_m_ (maximum quantum efficiency of photosystem II) in cold-stressed plants was over 7× greater when CaMV 35s was utilized as a promoter than when Ubi was utilized as a promoter (**Figure 7A**). The impact of stress duration (moderator) on the effect of *DREB* overexpression on *F*_v_/*F*_m_ (main effect) in *DREB* transgenic plants subjected to cold stress was most evident at a stress duration category of (4 day, 8 days). The impact average for all stress duration categories was 86, 219, 103, 32, 26, and 25% for other, (4 days, 8 days), (2 days, 4 days), (12 h, 24 h), (6 h, 12 h), and (0 h, 6 h), respectively (**Figure 7B**). Within the stress-severity moderator, chilling had an almost fivefold greater impact than the “other” level of stress severity (**Figure 7C**). In both the recipient-type category and donor-type category of moderators, a significantly larger effect was observed when a dicot was the recipient and donor of the *DREB* gene, than when the recipient and donor was a monocot (**Figures 7D,F**). The effect of *DREB* overexpression on *F*_v_/*F*_m_ was significantly greater, relative to other genera of donors, when the *DREB* gene donor was *Arabidopsis* (**Figure 7G**). *Arabidopsis* as a recipient, compared to the “other” category, however, slightly increased *F*_v_/*F*_m_ by 24% when transgenic plants were subjected to cold stressed conditions (**Figure 7E**).

### Activity of SOD and Soluble Sugar Content

A greater positive effect (126%) on SOD activity was observed when the stress durations was (2 days, 4 days) (**Figure 8A**). The impact of *DREB* overexpression on SOD activity was significantly greater when the stress severity was chilling (**Figure 8B**) rather than the “other” level of stress severity. The three variables within the recipient genus moderator had a similar effect on increasing SOD activity. The impact average for the recipient variables, *Chrysanthemum*, *Nicotiana*, and “other” species was 64%, 78%, and 60%, respectively (**Figure 8C**). *Arabidopsis* as a donor genus, also had a significant effect (64%) on increasing SOD activity (**Figure 8D**).

Regardless of the type of promoter (moderator) used to overexpress the DREB transcription factor, a combined average positive effect of 72% was observed on soluble sugar content (**Figure 9A**), although the effect of “other” promoters was not significant while the effect of the 35s promoter was highly significant. When the *DREB* recipient was *Arabidopsis*, the effect of *DREB* overexpression on soluble sugar content was twice as great when the recipient genus was *Arabidopsis*, than when the recipient was *Nicotiana* (**Figure 9B**). In the moderator category, donor -genus, *Arabidopsis* and “other species” had a similar influence on soluble sugar content in transgenic plants subjected to a cold stress (**Figure 9C**).

### Survival

When plants were not acclimated prior to being subjected to a cold stress, the effect of *DREB* overexpression on survival was over 5× greater than when plants were acclimated prior to the cold stress (**Figure 10A**). The impact of *DREB* overexpression on survival of transgenic plants subjected to a cold stress was about 2× greater when CaMV35S or Ubi was used as a promoter than when rd29a was the promoter (**Figure 10B**). In the moderator category, stress duration, the effect of *DREB* overexpression on survival was greatest (16-fold or 1,507%) when the stress duration was (2 days, 4 days) (**Figure 10C**). The impact of *DREB* overexpression on chilling stress resulted in the greatest level of survival among the variables listed under the moderator, stress severity, exhibiting an 11-fold (1085%) increase in survival, relative to the more modest increases observed in survival exhibited in response to a moderate or severe freezing stress (**Figure 10C**). The effect of *DREB* overexpression on survival was larger when the *DREB* donor and recipient were the same genus than when the donor and recipient were from different genera (**Figure 10E**). The impact of the moderator, recipient-type and donor-type on survival of *DREB* transgenic plants was similar. When the recipient and donor were dicots, however, the effect was slightly greater than when the recipients and donors were monocots (**Figures 10F,H**). In regard to recipient genus (a moderator), the effect of *DREB* overexpression on survival of transgenic plants averaged 333, 223, 203, 584, and 389% for *Arabidopsis, Hordeum*, *Nicotiana*, *Oryza*, and “other” species, respectively (**Figure 10G**). In regards to the donor genus moderator, a significant positive impact, averaging 518, 121, 251, 113, 238, and 244%, was observed for *Arabidopsis, Gossypium*, *Hordeum*, *Oryza*, *Triticum*, and “other” species, respectively (**Figure 10I**).

## Discussion

Cold hardiness in plants is a complex phenomenon, and is affected by temperature, day length, and a variety of plant parameters, such as maturity, water content, nutrition, physiology, and dormancy status (Gusta and Wisniewski, 2013). Plant adaptation to temperature stress involves various morphological, physiological, and biochemical changes, that are induced by the expression of transcription factors, target genes, gene product accumulation, and hormones. The present study utilized a meta-analysis approach to statistically quantified the extent to which the effect of overexpression of a DREB transcription factor affects several physiological indicators, including survival, and how that impact is altered by experimental parameters, such as the genus of the *DREB* donor or recipient, the promoter used to drive expression, the duration and severity of the stress, and whether the plant is acclimated or non-acclimated, etc.

Results indicated that overexpression of a *DREB* transcription factor in transgenic plants enhanced plant survival and decreased injury as measured by electrolyte leakage and MDA content. Many studies have utilized electrolyte leakage to measure freezing tolerance in transgenic and wild-type plants, where lower levels of electrolyte leakage indicate smaller degrees of injury (Polashock et al., 2010; Byun et al., 2015). The meta-analysis revealed that the effect of *DREB* overexpression on enhanced cold hardiness in transgenic plants is greater than in non-transgenic wild-type plants when control and transgenic plants are non-acclimated than when both categories of plants are acclimated. Most of the transgenic species are not chilling sensitive, so one would not expect a great deal of injury to occur when either transgenic or wild-type plants are exposed to low, non-freezing temperatures. The injury in wild-type plants would be less when they are acclimated compared to when they are non-acclimated. Hence the magnitude of the difference in injury levels, as measured by electrolyte leakage, between transgenic and wild-type plants would be greater when non-acclimated plants are compared vs. acclimated plants. The lowest level of electrolyte leakage would occur in response to low, non-freezing temperatures as *Arabidopsis* is not a chilling-sensitive species, so the impact of overexpressing a DREB transcription factor would be least evident when transgenic vs. wild-type plants are compared.

Malondialdehyde (MDA) is a product of membrane lipid peroxidation, and directly reflects the extent of membrane damage. The lower MDA content in *DREB* overexpressing plants indicates a higher stability of biological membrane systems relative to the membrane systems in non-transformed plants. The meta-analysis also indicated that the type of promoter used to drive *DREB* expression also has an impact on the degree to which parameters, such as electrolyte leakage and MDA content are affected. In addition to its impact on cold hardiness, the meta-analysis included four studies that examined the impact of overexpression of *DREB* on heat stress. Although the four papers provided evidence that over-expression of *DREB* in chrysanthemums, *Arabidopsis*, and *Nicotiana* significantly improved heat tolerance, this parameter has not been examined in other species (Hong et al., 2009; Lee et al., 2010; Kidokoro et al., 2015). The positive effect of *DREB* overexpression on heat tolerance revealed in our meta-analysis suggests that this aspect should be further investigated.

Superoxide dismutase is one of the key enzymes that plays a role in scavenging ROS (reactive oxygen species) in living cells (Yang et al., 2010). Increases in SOD activity could reduce the level of active-oxygen free radicals, which can cause injury to cells. When ROS levels are sufficiently high, plants suffer from oxidative stress. The meta-analysis indicated that SOD activity was higher in transgenic plants overexpressing *DREB* than in wild-type, non-transgenic plants. Chronic photoinhibition of photosystem II (PSII) may be induced in plants exposed to LT stress. This is because LT generally reduces the rate of biological reactions (mainly carbon dioxide reduction and photorespiration), thereby limiting the excitation energy of the sink for absorption (Allen and Ort, 2001). *F*_v_/*F*_m_, the ratio of variable to maximal chlorophyll fluorescence, is often used to measure the activity of photosystem II (PSII) and inhibition that is not rapidly reversible (Demmig-Adams et al., 1990). The impact of *DREB* overexpression on *F*_v_/*F*_m_ was observed to increase when plants were exposed to temperature stress, indicating that overexpression of *DREB* improves cold hardiness by allowing plants to maintain a higher photochemical efficiency and photosynthetic rate.

Soluble sugar can stabilize enzymes and cellular structures, and also increase the osmotic concentration of cell sap, thus lowering the freezing point (Krasensky and Jonak, 2012). On average, overexpression of *DREB* increased soluble sugar content when transgenic plants were not exposed to temperature stress, although the increase was not significant. *DREB* overexpression, however, did significantly increase soluble sugar content when transgenic plants were subjected to temperature stress. The increase in compatible solutes was more readily apparent with proline, where overexpression of *DREB* significantly increased proline in transgenic plants whether or not they were exposed to temperature stress (**Figures 1A,B**). The increase was impacted by the promoter of the gene, donor type, recipient type, and whether the same or different donor and recipient genera were used in the donor/recipient combination.

*DREB* expression constitutively driven by the CaMV 35s promoter has an immediate impact on regulating the expression of many functional genes and on signal transduction activity. Genes that are impacted include the expression of sucrose phosphate synthase and a proline synthase gene. Plants use energy to synthesize proline, sugar, and e other compatible solutes, and other accumulation of these substances can interfere with normal plant metabolism. This may be the reason why *DREB* overexpressing plants grow more slowly. Thus, this aspect of plant adaptation to abiotic stress requires further investigation.

Temperature plays a critical role in determining the rate and ability of a plant to photosynthesize effectively. The *F*_v_/*F*_m_ ratio (maximal quantum yield of PSII) is the primary component of photosynthesis that is negatively impacted by low-temperature stress, and can be used to evaluate photosynthetic capacity in response to abiotic stress (Krause and Weis, 1991; Ferrante and Maggiore, 2007). Photoinhibition of PSII was reported to be alleviated at chilling temperatures in *CBF1* transgenic plants (Jaglo et al., 2001; Hsieh et al., 2002). The meta-analysis also revealed that overexpression of *DREB* reduces damage to PSII, as measured by *F*_v_/*F*_m_, when transgenic plants are subjected to chilling stress.

A variety of metabolic reactions are inhibited when plants are subjected to LT stress, resulting in distinct phenotypic changes, the degree to which is impacted by the genetic background of the plant (Chinnusamy et al., 2007). The most characteristic effect of both low and HT stress on plants is decreased growth, as measured by plant height and plant survival. Meta-analysis confirmed that transgenic plants overexpressing DREB under non-stress conditions displayed a significant reduction in height resulting in a dwarf phenotype. This is most greatly impacted by the constitutive CaMV 35s promoter. The use of the CaMV 35s promoter to drive *DREB* overexpression resulted in dwarf growth phenotypes and delayed flowering in *Arabidopsis*, *Brassica napus*, and rice (Gilmour et al., 2000; Jaglo et al., 2001; Ito et al., 2006). The stress-inducible rd29A promoter is used by many researchers to reduce the negative effects of *DREB* expression on plant growth under non-stress conditions (Kasuga et al., 1999; Chen et al., 2009; Kidokoro et al., 2015). While reduction in plant growth and delayed flowering are negative effects for agronomic crops, they can be advantageous for turf grass. A dwarf, reduced-growth phenotype is an excellent trait for turf grass, because it would reduce the economic costs associated with mowing (Fry and Huang, 2004). Thus, the overexpression of *DREB* could potentially replace the use of plant growth inhibitors to reduce the height of turf grass and increase its resistance to temperature stress and thus warrants further research.

The increased survival evident in *DREB* transgenic plants is of special interest since it was the plant attribute most affected by overexpression by as much as 303%. The meta-analysis indicated that survival of transgenic plants overexpressing a *DREB* gene increased significantly, relative to wild-type plants, after exposure to either low-temperature or high-temperature stress, which most directly reflects the increased resistance of transgenic plants to temperature stress. The increased resistance of transgenic plants to low-temperature stress, relative to the wild-type plants, was most evident in plants that had not been cold acclimated, suggesting that *DREB* mimics natural LT acclimation. The physiological and biochemical processes associated with cold acclimation in fact have been demonstrated to involve *DREB* and cold regulated (*COR*) genes expression, and these same processes are induced in *DREB* overexpressing plants (Thomashow, 1999; Chen et al., 2014). The improved low-temperature tolerance is also indicated by reduced levels of electrolyte leakage in transgenic plants (**Figure 5A**).

Stress severity and stress duration also had a significant impact on survival. The effect of overexpressing *DREB* on survival was greater when plants were exposed to a chilling stress than when they were exposed to a more severe stress. And the effect of the level of stress becomes even greater with the increase in stress duration, until reaching a maximum after which there was a subsequent decrease in survival as stress duration continued to increase. These results demonstrate that the impact of *DREB* expression on temperature stress resistance is complex, and that a full understanding will require further research. When the accumulation of compatible solutes reaches its limit, as likely occurs when plants are exposed to a severe stress or moderate longer-term stress, the impact of *DREB* overexpression is decreased. Maximum cold hardiness, however, is often not as crucial for survival, as the level of temperature tolerance that is determined by factors such as the timing and rate of cold acclimation, retention of cold hardiness, the timing and rate of dehardening, and the ability to reharden (Gusta and Wisniewski, 2013). This may explain why the impact of overexpression of *DREB/CBF* genes varies greatly in the different studies.

In regard to donor genus or recipient genus, *A. thaliana* was the most studied species. *DREB* genes were first transferred into *A. thaliana* and their overexpression was demonstrated to enhance both freezing (Jaglo-Ottosen et al., 1998) and drought tolerance (Liu et al., 1998). The impact of this gene family when it was obtained from species other than *Arabidopsis*, has still been typically studied in transgenic *Arabidopsis* (Gilmour et al., 2004; Park et al., 2015) often because of convenience, the amount of information available in *Arabidopsis*, and because a transformation system for the species that served as a source for the *DREB* gene was unavailable. Although the trend for the impact of over expression of *DREB* in *Arabidopsis* compared to the other species is the same, the degree is different. Meta-analysis indicated that the identity of the donor and recipient plant influenced the impact that *DREB* expression had on each effect size in transgenic plants. Therefore, the variability in the effect of *Arabidopsis* in *DREB* overexpression studies raises the question as to it can serve as a model system for evaluating the impact of *DREB* overexpression cold tolerance. In addition, when the donor and recipient genus were same, the effect of *DREB* overexpression was greater than when the donor and recipient were different species. Dubouzet et al. (2003) reported that the *Arabidopsis* DREB1A protein binds to both ACCGAC and GCCGAC at the same efficiency, but that the OsDREB1A (OsDREB1A from *Oryza sativa*) protein prefers GCCGAC to ACCGAC as a binding site. Differences in the promoter sequence of *DREB* target genes, and differences in *DREB* members in different species may also contribute to the different phenotypes that have been observed.

The meta-analysis conducted in the present study revealed several factors that require further clarification and/or study. The number of studies that evaluated the effect of overexpression under non-stressed conditions and in response to stress is much less than the number that only investigated the response to stress. In addition, detailed information about the specific *DREB* gene used is lacking in some studies. Biological research has entered the era of ‘big data,’ and the plant science community will require access to a Big-Data compatible parallel computing and data management infrastructure. There is also a need to investigate and/or develop analytic programs that are capable of extracting information from the overwhelming amounts of data that are currently available and are being generated on a daily basis (Ma et al., 2014). We suggest that a standardized set of information be provided in each published study that will allow others to utilize the information in larger sets of data analytics. In conclusion, a meta-analysis of the impact of *DREB* overexpression on temperature stress (primarily low-temperature stress) utilizing hundreds of studies revealed a positive effect. The meta-analysis also provided a clearer picture of what components are affected (main variables) and how various parameters (modulators) of a study, such as the source and recipient of the *DREB* transgene, the promoter used, the duration and severity of the stress, etc., impacted each of the main effects. The analysis clearly revealed the valuable information that can be obtained when meta-analysis is utilized to examine the impact of overexpression of a transgene in cases where numerous studies have been conducted. Thus, it may be beneficial to apply meta-analysis to other transgene studies, where overexpression or silencing has been demonstrated to impact disease resistance, ripening, and quality traits, such as color, taste, or firmness.

## Author Contributions

Z-MC designed the study. CD performed the research and wrote the paper. YM analyzed the data. DZ and MW were involved in writing the manuscript.

## Conflict of Interest Statement

The authors declare that the research was conducted in the absence of any commercial or financial relationships that could be construed as a potential conflict of interest.

## Footnotes

1 http://getdata-graph-digitizer.com

## Supplementary Material

DATA SHEET S1 Supporting information 1.
